# Urinary Excretion of *N*^1^-Methylnicotinamide and *N*^1^-Methyl-2-Pyridone-5-Carboxamide and Mortality in Kidney Transplant Recipients

**DOI:** 10.3390/nu12072059

**Published:** 2020-07-10

**Authors:** Carolien P.J. Deen, Anna van der Veen, António W. Gomes-Neto, Johanna M. Geleijnse, Karin J. Borgonjen-van den Berg, M. Rebecca Heiner-Fokkema, Ido P. Kema, Stephan J.L. Bakker

**Affiliations:** 1 Department of Internal Medicine, University of Groningen, University Medical Center Groningen, 9713 GZ Groningen, The Netherlands; a.w.gomes.neto@umcg.nl (A.W.G.-N.); s.j.l.bakker@umcg.nl (S.J.L.B.); 2 Department of Laboratory Medicine, University of Groningen, University Medical Center Groningen, 9713 GZ Groningen, The Netherlands; a.van.der.veen03@umcg.nl (A.v.d.V.); m.r.heiner@umcg.nl (M.R.H.-F.); i.p.kema@umcg.nl (I.P.K.); 3 Top Institute of Food and Nutrition (TIFN), 6709 PA Wageningen, The Netherlands; 4 TransplantLines Food and Nutrition Biobank and Cohort Study, University of Groningen, University Medical Center Groningen, 9713 GZ Groningen, The Netherlands; 5 Division of Human Nutrition and Health, Wageningen University, 6708 PB Wageningen, The Netherlands; marianne.geleijnse@wur.nl (J.M.G.); karin.borgonjen@wur.nl (K.J.B.-v.d.B.)

**Keywords:** *N*^1^-methylnicotinamide, *N*^1^-methyl-2-pyridone-5-carboxamide, urinary excretion, niacin status, renal transplantation, mortality, vitamin B_3_, tryptophan, dietary intake

## Abstract

It is unclear whether niacin nutritional status is a target for improvement of long-term outcome after renal transplantation. The 24-h urinary excretion of *N*^1^-methylnicotinamide (*N*^1^-MN), as a biomarker of niacin status, has previously been shown to be negatively associated with premature mortality in kidney transplant recipients (KTR). However, recent evidence implies higher enzymatic conversion of *N*^1^-MN to *N*^1^-methyl-2-pyridone-5-carboxamide (2Py) in KTR, therefore the need exists for interpretation of both *N*^1^-MN and 2Py excretion for niacin status assessment. We assessed niacin status by means of the 24-h urinary excretion of the sum of *N*^1^-MN and 2Py (*N*^1^-MN + 2Py), and its associations with risk of premature mortality in KTR. *N*^1^-MN + 2Py excretion was measured in a longitudinal cohort of 660 KTR with LS-MS/MS. Prospective associations of *N*^1^-MN + 2Py excretion were investigated with Cox regression analyses. Median *N*^1^-MN + 2Py excretion was 198.3 (155.9–269.4) µmol/day. During follow-up of 5.4 (4.7–6.1) years, 143 KTR died, of whom 40 due to an infectious disease. *N*^1^-MN + 2Py excretion was negatively associated with risk of all-cause mortality (HR 0.61; 95% CI 0.47–0.79; *p* < 0.001), and infectious mortality specifically (HR 0.47; 95% CI 0.29–0.75; *p* = 0.002), independent of potential confounders. Secondary analyses showed effect modification of hs-CRP on the negative prospective association of *N*^1^-MN + 2Py excretion, and sensitivity analyses showed negative and independent associations of *N*^1^-MN and 2Py excretion with risk of all-cause mortality separately. These findings add further evidence to niacin status as a target for nutritional strategies for improvement of long-term outcome in KTR.

## 1. Introduction

The treatment of choice for end-stage renal disease is renal transplantation, with one-year patient survival exceeding 90% [1,2]. Despite advances in short-term outcome, kidney transplant recipients (KTR) remain at highly increased risk of premature mortality compared to the general population [3,4]. Nutrition is increasingly acknowledged as a modifiable factor to improve prospects in KTR [5]. Many factors, such as dietary restrictions, stress, medication use, and comorbidities, pose a challenge to maintain adequate nutrition after renal transplantation [5,6,7,8,9,10], while adequate nutrition has been implicated to prevent clinical conditions that adversely affect long-term outcome and premature mortality in KTR [11,12,13,14,15,16,17].

**Figure 1:**
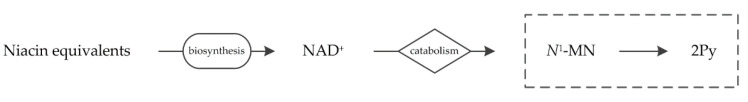
Schematic overview of biosynthesis of NAD^+^ from niacin equivalents, and catabolism of NAD^+^ via formation of *N*^1^-MN and 2Py, respectively, framed by the dotted line. *N*^1^-methylnicotinamide; 2Py, *N*^1^-methyl-2-pyridone-5-carboxamide.

Niacin status is a potential target for improvement of long-term outcome in KTR. Niacin, or vitamin B_3_, is the precursor of the nicotinamide nucleotide coenzyme NAD^+^. An adequate niacin status is pivotal to supply substrates of NAD^+^-consuming enzymes, and reducing equivalents for energy metabolism [18]. NAD^+^ catabolism proceeds via formation of *N*^1^-methylnicotinamide (*N*^1^-MN) and *N*^1^-methyl-2-pyridone-5-carboxamide (2Py), respectively, and both products are excreted in urine (Figure 1) [19]. Although 2Py is the final product of NAD^+^ catabolism, the 24-h urinary excretion of *N*^1^-MN is the most common and recommended index of niacin nutritional status [20,21]. Importantly, lower *N*^1^-MN excretion has shown to be associated with higher risk of premature all-cause mortality in KTR [22]. Further evidence on the potential of niacin nutrition for improvement of long-term outcome in KTR is unrevealed, as previous studies have only addressed pharmacological doses of niacin within the context of chronic kidney disease and renal transplantation [23,24,25,26,27,28], and dietary intake of niacin to reduce frailty and risk of mortality in elderly [29].

In a recent study, we have demonstrated lower *N*^1^-MN excretion to be paralleled by higher 2Py excretion in KTR, due to putative increased enzymatic conversion of *N*^1^-MN to 2Py in conditions of renal function impairment [30]. Accordingly, it has been suggested that interpretation of *N*^1^-MN excretion alone is of limited value, at least in conditions of renal function impairment, and 2Py should be additionally interpreted for assessment of niacin status [30].

At present, it is unclear whether niacin status is a potential target for nutritional strategies for improvement of long-term outcome after renal transplantation. As the presence of increased enzymatic conversion of *N*^1^-MN to 2Py is presumed in KTR, it remains to be determined whether prospective associations of niacin status with premature all-cause mortality exist when taking into account both *N*^1^-MN and 2Py excretion, and therefore can be fully attributed to niacin status, rather than renal function and other factors that may affect the conversion of *N*^1^-MN to 2Py. Prospective associations of niacin status may be of particular interest within the context of infectious diseases as a leading cause of premature mortality in KTR, given the reported benefits of niacin on the inflammatory response during infections [31,32,33,34]. Therefore, the primary aim of this study is to assess the 24-h urinary excretion of the sum of *N*^1^-MN and 2Py (*N*^1^-MN + 2Py) in KTR, and to prospectively investigate whether niacin nutritional status is associated with risk of all-cause mortality in KTR. The secondary aim of this study is to prospectively investigate whether niacin nutritional status is associated with risk of infectious mortality in KTR.

## 2. Materials and Methods

### 2.1. Study Population

This longitudinal study was conducted in a single-center cohort of 707 KTR (≥18 years) who visited the outpatient clinic of the University Medical Center Groningen (Groningen, The Netherlands) between November 2008 and June 2011. All KTR had a stable graft for at least one year, and none had a history of drug and/or alcohol abuse [35,36,37]. Exclusion of subjects with use of niacin supplements or missing biomaterial left 660 RTR eligible for statistical analyses. All participating subjects provided written informed consent. The institutional review board (METc 2008/186) approved the study protocol according to the principles of the Declaration of Helsinki.

### 2.2. Data Collection

Baseline data were gathered during morning visits to the outpatient clinic. Subjects collected a 24-h urine sample according to strict protocol prior to their visit. In this protocol, subjects were instructed to discard the first morning void, to collect all subsequent urine throughout the next 24 h, and to include the next first morning void of the day of the visit to the outpatient clinic. Upon completion of the 24-h urine sample collection, fasting blood samples were drawn. Directly after sample collection, laboratory measurements were performed with routine clinical laboratory assays based on spectrophotometry (Roche Diagnostics, Rotkreuz, Switzerland). Samples were stored at −80 °C. Parameters on hemodynamics and body composition were measured according to a protocol described previously [35]. Proteinuria was defined as total urinary protein excretion of ≥0.5 g/day. Diabetes was defined as use of antidiabetic medication or fasting plasma glucose of ≥7.0 mmol/L. Proteinuria was defined as total urinary protein excretion of ≥0.5 g/day. As KTR have previously been reported to be commonly deficient in vitamin B_6_ [12] as an essential cofactor in de novo biosynthesis of niacin [38], vitamin B_6_ status was assessed by means of plasma vitamin B_6_ as its principal, metabolically active form pyridoxal-5′-phosphate using a high-performance liquid chromatography method (Waters Alliance, Milford, MA, USA) with fluorescence detection (JASCO, Inc., Easton, MD, USA) [12].

Semiquantitative food frequency questionnaires (FFQs), validated for KTR as reported previously [36], were used for assessment of dietary intake [39,40]. The questionnaires, inquiring on 177 food items over the last month, were self-administered and filled out at home. FFQs were checked for completeness and consistency by trained researchers during the visit to the outpatient clinic [36]. The Dutch Food Composition Table of 2006 was used to convert dietary data into daily nutrient intake [41]. Intake of niacin equivalents was calculated by adding up the intakes of niacin and one-sixtieth of tryptophan. Subjects using niacin supplements were excluded. Separate questionnaires were used for assessment of smoking behavior [11]. Medical records were used to obtain data on medical history, and use of vitamin supplements and medication [11].

The combined creatinine and cystatin C-based Chronic Kidney Disease Epidemiology Collaboration equation was used for calculation of the estimated glomerular filtration rate (eGFR) [42], being the most accurate equation in KTR [43].

### 2.3. Assessment of N^1^-MN and 2Py Excretion

*N*^1^-MN and 2Py concentrations were measured with a validated liquid chromatography (Luna HILIC column; Phenomenex, Torrance, CA, USA) isotope dilution-tandem mass spectrometry (Quattro Premier; Waters, Milford, MA, USA) (LC-MS/MS) method, as reported previously [22,30,44]. The 24-h urinary excretion of *N*^1^-MN and 2Py (μmol/day) was calculated by multiplying concentrations (μmol/L) by total urine volume calculated from weight (L/day).

### 2.4. Clinical Endpoints

The primary endpoints of this study were all-cause mortality and infectious mortality, and the secondary endpoint was noninfectious mortality. Infectious mortality was defined as death due to an infectious disease according to the International Classification of Diseases, Ninth Revision (ICD-9) codes 001–139. Noninfectious mortality was defined as death due to cardiovascular, malignant, or other (miscellaneous) diseases. Information on survival status and causes of death was obtained by linking the patient number to the database of the Central Bureau of Statistics and then cause of death as reported by physicians. Up-to-date information on survival status and causes of death was ensured through the continuous surveillance system of the outpatient program [17]. Endpoints were recorded until 30 September 2015 with no loss of subjects to follow-up.

### 2.5. Statistical Analysis

Normally distributed, skewed, and nominal data are presented as the mean ± SD, median (interquartile range (IQR)), and absolute number (percentage), respectively. The corresponding frequency distribution and Q-Q plots were visually judged to check for normality assumptions.

For cross-sectional analyses, baseline characteristics of KTR were divided into tertiles of *N*^1^-MN + 2Py excretion stratified by sex (T1, T2, and T3). Linear regression analyses were used to investigate associations of 2-base log-transformed *N*^1^-MN + 2Py excretion with baseline characteristics, with adjustment for sex. Pearson correlation was used to investigate the correlation of *N*^1^-MN + 2Py excretion with dietary intake of niacin equivalents.

For prospective analyses, Kaplan–Meier curves were plotted with log-rank tests to estimate the difference between sex-stratified tertiles of *N*^1^-MN + 2Py excretion for all-cause mortality, infectious mortality, and noninfectious mortality outcomes. Subsequently, Cox proportional hazards regression models were fitted to *N*^1^-MN + 2Py excretion as a continuous variable adjusted for sex (model 1) for all-cause mortality, infectious mortality, and noninfectious mortality, as well as a sex-stratified tertile-based categorical variable for all-cause mortality. Potential confounders were included as covariates in regression models to control for confounding. Crude associations were adjusted cumulatively for age and body surface area (model 2) and, in subsequent models, additively for high-sensitivity C-reactive protein (hs-CRP) (model 3), plasma vitamin B_6_ (model 4), renal function parameters (model 5), medication use (model 6), and intake of alcohol and energy (model 7). Potential confounders were included additive rather than cumulative to prevent overfitting by inclusion of a larger number of variables in a single model than allowed for by the number of outcome events during prospective follow-up. By inclusion of a maximum number of six variables with regard to the number of infectious mortality events (*n* = 40) as a primary outcome, the Cox regression models comply with the rule of thumb for the number of outcome events per variable that is set at a minimum of 5 to preferably 20 outcome events per variable [45,46,47]. Variables that could lie in the causal pathway of *N*^1^-MN + 2Py excretion and all-cause mortality were not included in regression models, because adjustment for potential mediators might introduce bias in the estimation of the total effect of exposure on outcome [48]. Kaplan–Meier plots of the survival and the log-survival function entering the sex-stratified *N*^1^-MN + 2Py excretion tertile group variable were visually judged to check for the proportionality of hazards and the linearity of log-hazards assumptions.

In secondary prospective analyses, effect modification was assessed by including the cross product term of each potential confounder included in models 2–7 and 2-base log-transformed *N*^1^-MN + 2Py excretion in the Cox regression model adjusted for sex (model 1). Subsequent stratified analyses were performed for subgroups of significant effect modifiers on the associations of *N*^1^-MN + 2Py excretion with premature mortality. In addition, potential associations of *N*^1^-MN + 2Py excretion with death due to cardiovascular, malignant, and other (miscellaneous) diseases were separately investigated in addition to noninfectious mortality as a whole. Finally, possible nonlinearity of associations of *N*^1^-MN + 2Py excretion with all-cause mortality and infectious mortality was investigated by including the quadratic and cubic terms of *N*^1^-MN + 2Py excretion in addition to its linear term in the Cox regression models 1–7.

For sensitivity analyses, the 24-h urinary excretion of *N*^1^-MN and 2Py, urinary ratio of 2Py to *N*^1^-MN (2Py/*N*^1^-MN), and dietary intake of niacin equivalents were separately assessed, and prospectively investigated for associations with risk of all-cause mortality in Cox regression analyses. In further sensitivity analyses, the first half of KTR that died during follow-up (i.e., during ≤3.17 years of follow-up; *n* = 72 events) was excluded for assessment of the association of *N*^1^-MN + 2Py excretion with risk of all-cause mortality and infectious mortality in Cox regression analyses, to make it unlikely that associations of *N*^1^-MN + 2Py excretion with outcome are driven by reverse causation. Finally, both in the full cohort and the subgroup of KTR that died during >3.17 years of follow-up (*n* = 71 events), the association of *N*^1^-MN + 2Py excretion with risk of all-cause mortality and infectious mortality was adjusted cumulatively for sex; age; body surface area; use of proliferation inhibitors, acetylsalicylic acid, and proton pump inhibitors; intake of alcohol and energy in Cox regression analyses.

Statistical significance was considered to be indicated by a two-sided *p*-value of less than 0.05 and SPSS Statistics version 23.0 (IBM, Armonk, NY, USA) was used as software for all statistical analyses.

## 3. Results

### 3.1. Baseline Characteristics and Cross-Sectional Analyses

Baseline characteristics of KTR across sex-stratified tertiles of *N*^1^-MN + 2Py excretion (M: 181.3, 181.3–261.2, and >261.2 μmol/day; F: <147.7, 147.7–216.9, and >216.9 μmol/day in T1, T2, and T3, respectively) are shown in Table 1. At inclusion (5.6 (2.0–12.0) years after transplantation), mean age was 53.0 ± 12.7 years, and 57% were male. Median *N*^1^-MN + 2Py excretion was 198.3 (155.9–269.4) µmol/day.

Sex-adjusted associations of *N*^1^-MN + 2Py excretion with baseline characteristics are shown in Table 1. Body mass index (BMI), body surface area, alcohol consumption, plasma vitamin B_6_, high-sensitivity C-reactive protein (hs-CRP), eGFR, and use of proliferation inhibitors were positively associated with *N*^1^-MN + 2Py excretion, while age and use of acetylsalicylic acid and proton pump inhibitors were negatively associated with *N*^1^-MN + 2Py excretion. Medications that are known to potentially affect niacin status, including cyclosporine, azathioprine, and anticonvulsants, used by 253 (38%), 112 (17%), and 19 (3%) of KTR, respectively, were not associated with *N*^1^-MN + 2Py excretion. *N*^1^-MN + 2Py excretion was positively correlated with dietary intake of niacin equivalents (*r* = 0.23; *p* < 0.001).

### 3.2. Primary Prospective Analyses

**Figure 2:**
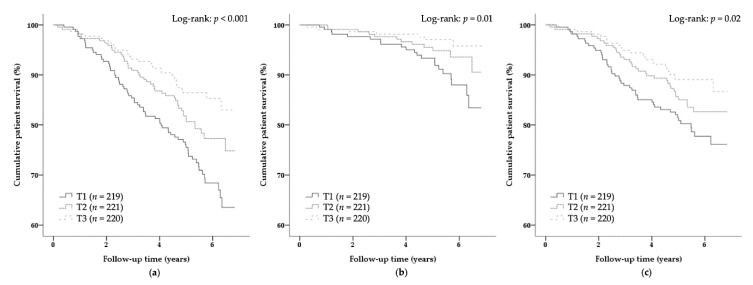
Kaplan–Meier survival curves with log-rank tests for (**a**) all-cause mortality, (**b**) infectious mortality, and (**c**) noninfectious mortality in KTR according to sex-stratified tertiles of *N*^1^-MN + 2Py excretion. *N*^1^-MN + 2Py excretion was <181.3, 181.3–261.2, and >261.2 μmol/day for males, and <147.7, 147.7–216.9, and >216.9 μmol/day for females in T1, T2, and T3, respectively. *N*^1^-MN, N1-methylnicotinamide; KTR, kidney transplant recipients; 2Py, *N*^1^-methyl-2-pyridone-5-carboxamide.

During a median follow-up time of 5.4 (4.7–6.1) years, 143 (22%) KTR died, of whom 40 (6%) due to an infectious disease, and 103 (16%) due to noninfectious diseases, comprising cardiovascular (56 (9%)), malignant (26 (4%)), and other (miscellaneous) diseases (21 (3%)). Survival curves according to sex-stratified tertiles of *N*^1^-MN + 2Py excretion for all-cause mortality, infectious mortality, and noninfectious mortality are shown in Figure 2. Rates of all-cause mortality, infectious mortality, and noninfectious mortality increased with decreasing sex-stratified tertiles of *N*^1^-MN + 2Py excretion (Log-rank: *p* < 0.001, *p* = 0.01, and *p* = 0.02, respectively) (Figure 2). Cox regression analyses exposed a negative association of *N*^1^-MN + 2Py excretion with all-cause mortality (Model 2: HR 0.61; 95% CI 0.47–0.79; *p* < 0.001), and higher risk of all-cause mortality for KTR in the lowest (T1) and middle sex-stratified tertiles of *N*^1^-MN + 2Py excretion (T2) compared to those in the highest tertile (T3) as reference (Model 2: HR 2.03; 95% CI 1.31–3.15; *p* = 0.002 and HR 1.45; 95% CI 0.92–2.30; *p* = 0.11, respectively) (Table 2). Cox regression analyses furthermore exposed a negative association of *N*^1^-MN + 2Py excretion with infectious mortality (Model 2: HR 0.47; 95% CI 0.29–0.75; *p* = 0.002) (Table 3). Prospective associations with all-cause mortality and infectious mortality, and less so noninfectious mortality, were independent of potential confounders, including sex, age, body surface area, plasma vitamin B_6_, renal function parameters, medication use, and intake of alcohol and energy.

### 3.3. Secondary Prospective Analyses

Secondary analyses revealed significant effect modification of hs-CRP on the negative association of *N*^1^-MN + 2Py excretion with all-cause mortality (*p* = 0.02). Given the significant interaction of the association of *N*^1^-MN + 2Py excretion with risk of all-cause mortality for hs-CRP, subsequent stratified analyses for subjects in subgroups of hs-CRP were performed. The negative association of *N*^1^-MN + 2Py excretion with all-cause mortality was clearly present for subjects in the subgroup with serum hs-CRP ≤3 mg/L (Model 2: HR 0.48; 95% CI 0.34–0.68; *p* < 0.001), but to a lesser extent in the subgroup with serum hs-CRP >3 mg/L (Model 2: HR 0.79; 95% CI 0.52–1.20; *p* = 0.27) (Table 4). The same held for the negative association of *N*^1^-MN + 2Py excretion with infectious mortality, being clearly present for subjects in the subgroup with serum hs-CRP ≤3 mg/L (Model 2: HR 0.38; 95% CI 0.21–0.67; *p* = 0.001), but to a lesser extent in the subgroup with serum hs-CRP >3 mg/L (Model 2: HR 0.71; 95% CI 0.30–1.65; *p* = 0.43) (Table 4), and less so with noninfectious mortality. In addition, prospective associations of *N*^1^-MN + 2Py excretion with cardiovascular mortality, malignant mortality, and other (miscellaneous) mortality were less clearly present (Table S1). Moreover, *N*^1^-MN + 2Py excretion was associated with all-cause mortality and infectious mortality in a linear fashion, rather than a nonlinear fashion (all *p* > 0.05) (Table S2).

### 3.4. Sensitivity Analyses

The 24-h urinary excretion of *N*^1^-MN and 2Py, urinary 2Py/*N*^1^-MN, and dietary intake of niacin equivalents, and associations with risk of all-cause mortality are shown in Table 5 and Table 6, respectively. The 24-h urinary excretion of *N*^1^-MN and 2Py were 22.0 (15.8–31.8) µmol/day and 178.1 (130.3–242.8) µmol/day, respectively (Table 5). Urinary 2Py/*N*^1^-MN was 8.7 ± 3.8. Dietary intake of niacin equivalents was 35.6 ± 9.2 mg/day. The minimum recommended daily intake of niacin equivalents of 6.6 niacin equivalents per 1000 kcal is complied with by all KTR (≥9.6 mg/1000 kcal, respectively) [21]. Sensitivity analyses revealed the presence of independent prospective associations of the 24-h urinary excretion of *N*^1^-MN and 2Py separately (Model 2: HR 0.57; 95% CI 0.46–0.72; *p* < 0.001 and HR 0.65; 95% CI 0.51–0.84; *p* = 0.001, respectively) (Table 6), both in line with the findings on *N*^1^-MN + 2Py excretion (Table 2). Prospective associations of urinary 2Py/*N*^1^-MN and dietary intake of niacin equivalents with all-cause mortality were dependent on renal function parameters (Model 5: HR 1.00; 95% CI 0.95–1.04; *p* = 0.85) and intake of alcohol and energy (Model 7: HR 0.77; 95% CI 0.43–1.38; *p* = 0.38) (Table 6). When separately adjusted for in sensitivity analyses, prospective associations of urinary 2Py/*N*^1^-MN and dietary intake of niacin equivalents with all-cause mortality were mainly dependent on eGFR (Model 5: HR 1.00; 95% CI 0.95–1.05; *p* = 0.94), rather than proteinuria (Model 5: HR 1.05; 95% CI 1.01–1.09; *p* = 0.02) and primary renal disease (Model 5: HR 1.06; 95% CI 1.02–1.10; *p* = 0.007), and intake of energy (Model 7: HR 0.98; 95% CI 0.94–1.01; *p* = 0.18), rather than intake of alcohol (Model 7: HR 0.98; 95% CI 0.95–1.00; *p* = 0.04), respectively.

Prospective associations of *N*^1^-MN + 2Py excretion with all-cause mortality and infectious mortality remained present in the subgroup of KTP that died during >3.17 years of follow-up (Model 2: HR 0.56; 95% CI 0.39–0.82; *p* = 0.002 and HR 0.28; 95% CI 0.14–0.55; *p* < 0.001, respectively) (Table S3), indicating robustness of the present findings. Finally, prospective associations of *N*^1^-MN + 2Py excretion remained present independent of cumulative adjustment for sex; age; body surface area; use of proliferation inhibitors, acetylsalicylic acid, and proton pump inhibitors; intake of alcohol and energy for all-cause mortality (HR 0.75; 95% CI 0.56–0.99; *p* = 0.04) and, to a lesser extent, infectious mortality (HR 0.62; 95% CI 0.37–1.04; *p* = 0.07) in the full cohort of KTR; and for both all-cause mortality (HR 0.68; 95% CI 0.46–1.00; *p* = 0.05) and infectious mortality (HR 0.40; 95% CI 0.19–0.84; *p* = 0.02) in the subgroup of KTR that died during >3.17 years of follow-up.

## 4. Discussion

To the best of our knowledge, this is the first study to assess and prospectively investigate niacin nutritional status by means of the 24-h urinary excretion of the sum of *N*^1^-MN and 2Py in stable KTR. In cross-sectional analyses, we assessed the 24-h urinary excretion of *N*^1^-MN + 2Py. In prospective analyses, we investigated associations of niacin nutritional status with risk of all-cause mortality, and infectious mortality specifically. Importantly, we found that the 24-h urinary excretion of *N*^1^-MN + 2Py was negatively associated with higher risk of all-cause mortality, and infectious mortality specifically, independent of potential confounders adjusted for in separate models. Secondary analyses revealed effect modification of hs-CRP on the negative prospective association of *N*^1^-MN + 2Py excretion, and sensitivity analyses revealed negative and independent associations of *N*^1^-MN and 2Py excretion with all-cause mortality separately.

The potential of niacin nutrition for improvement of long-term outcome relies on the assessment of niacin status rather than dietary intake of niacin equivalents due to their differing bioavailability as NAD^+^ precursors [49]. In current literature and guidelines, the 24-h urinary excretion of *N*^1^-MN as a breakdown product of NAD^+^ is conceived the gold standard biomarker for assessment of niacin status [20,21]. Considerable evidence has, however, implied this biomarker to be responsive to multiple physiological and pathological factors [50,51,52,53,54,55], and most recently to renal function [30], and noted opposing shifts of 2Py excretion in parallel to *N*^1^-MN excretion [30,54]. Given the furthermore presumed presence of increased enzymatic conversion of *N*^1^-MN to 2Py in conditions of renal function impairment [30,55,56], additional interpretation of 2Py, rather than *N*^1^-MN alone, is indicated to address niacin status in KTR. Noteworthy, other conditions inherent to renal function decline, such as aging [57,58], may superimpose the latter proposition. In the present study, prospective associations of urinary 2Py/*N*^1^-MN and dietary intake of niacin equivalents were indeed confounded by renal function parameters (eGFR) and dietary intake (energy), respectively, while those of the urinary excretion of *N*^1^-MN and 2Py separately aligned with the urinary excretion of *N*^1^-MN + 2Py and therefore support the present conclusions.

Prospective studies on niacin nutrition are scarce, as the prevailing dietary intake of niacin equivalents is considered to meet the baseline requirements for NAD^+^ synthesis in developed countries [29,59]. Niacin equivalents comprise the root substrates of NAD^+^ biosynthetic pathways, being nicotinamide, nicotinic acid, and nicotinamide riboside for salvage pathways, and tryptophan for the de novo nicotinamide pathway [31]. There is a substantial body of evidence, however, that greater NAD^+^ availability from its dietary sources may be beneficial in various pathological conditions [18], including those that precipitate premature mortality among KTR [60,61]. Accordingly, the 24-h urinary excretion of *N*^1^-MN as breakdown product of NAD^+^ has shown to be negatively associated with premature all-cause mortality in KTR [22]. In the present study, this association remains when taking into account 2Py as the consecutive and final breakdown product of NAD^+^ additionally, and may therefore be solely attributed to niacin status.

The adherence to the recommended daily intake of niacin equivalents is undisputed in KTR. NAD^+^ availability from its dietary sources is, however, subject to secondary factors that may interfere with the enzymatic activities of NAD^+^ metabolic pathways. Indeed, the profound cross-sectional associations of *N*^1^-MN + 2Py excretion with vitamin B_6_ status and alcohol consumption are most likely explained by their implication in the de novo nicotinamide pathway [19] and NAD^+^ catabolism [62,63], respectively. Of note, these secondary factors did not appear to underlie the prospective associations of niacin status in KTR.

Evidently, the protective effect of niacin status on premature mortality in KTR is most likely explained by its ability to prompt NAD^+^ biosynthesis. In fact, NAD^+^ homeostasis has been linked to increased resistance to diseases that are the main contributors to premature mortality in KTR: cardiovascular, infectious, and malignant diseases [60,61]. In the present cohort, the preponderance of cardiovascular, infectious, and malignant diseases as leading causes of premature mortality after renal transplantation is unabated, accounting for 56 (40%), 40 (28%), and 26 (18%) out of 143 mortality cases separately, respectively, adding up to 122 (85%) collectively. Furthermore, within the context of renal diseases, beneficial effects of niacin in pharmacological doses have at least partly been explained by its native function to fuel NAD^+^ biosynthesis in previous studies [31].

In pathological conditions, NAD^+^ is presumed to drive benefits by its role as redox coenzyme, but also by the reciprocal activities of NAD^+^-consuming enzymes: sirtuins, poly(ADP-ribose) polymerases (PARPs), and cyclic ADP ribose synthases (CD38 and CD157). Sirtuins are implicated in longevity and protection of organs including the kidneys specifically, via deacetylation of factors related to apoptosis, senescence, and inflammation [64]. PARPs, however, deplete NAD^+^ to the point of impeding sirtuin activity in response to immune-related oxidative (DNA) damage [31]. Given the predominance of inflammatory and oxidative stress in KTR [65], higher niacin status may preserve NAD^+^-dependent sirtuin activity as a protective factor in this population.

The reciprocal activities of NAD^+^-consuming enzymes may also hold for the negative association of *N*^1^-MN + 2Py excretion with risk of infectious mortality specifically. Sirtuins inhibit proinflammatory factors, such as NF-kB and p53 [66], which is key in the supposed antioxidant and anti-inflammatory properties of niacin during infections [32,33,34]. Oppositely, PARPs not only attenuate anti-inflammatory sirtuin activity by limiting NAD^+^ levels, but also activate proinflammatory factors directly [67]. Benefits of niacin on the inflammatory response and survival have been exemplified during sepsis, and were likewise attributed to downregulation of intracellular signaling [32,33,34], mediated by sirtuins.

Alterations in NAD^+^ homeostasis may be apparent due to the prevalence of low-grade chronic inflammation in KTR. Tryptophan is quantitatively the most important NAD^+^ precursor, and its flux through the nicotinamide pathway is upregulated by indoleamine 2,3-dioxygenase in response to inflammatory cytokines during chronic inflammation [19]. Indeed, we found *N*^1^-MN + 2Py excretion to be positively associated with serum hs-CRP as a biomarker of low-grade chronic inflammation. In view of this, one can speculate upon tryptophan being shunted away from other pathways that use tryptophan as a precursor, including protein biosynthesis, to fuel NAD^+^ biosynthesis [19,68]. Such a shift may amplify pre-existing protein catabolism and negative protein balance [7,9], and accordingly add to the risk of premature mortality with lower niacin status in KTR. Therefore, although the protective effect of niacin status on mortality is stronger for individuals in the subgroup with lower serum hs-CPR in the present study, future studies may point out whether those in the subgroup with higher serum hs-CRP will profit from niacin nutrition exceeding their present status.

Strengths of the present study include the large sample size of KTR, the long follow-up time, and the comprehensive characterization of KTR to control for confounding and effect modification. In addition, 24-h urine collection takes into account the large diurnal variation of NAD^+^ catabolism [69,70], rather than single time-point sampling such as single-void urine or plasma collection. Importantly, the assessment of 2Py excretion in addition to *N*^1^-MN excretion by means of the 24-h urinary excretion of *N*^1^-MN + 2Py eliminates the presence of increased conversion of *N*^1^-MN to underlie the prospective associations of niacin status in KTR. Limitations of this study include its observational nature which inherently prohibits causal inferences. Similarly, the observational nature of this study precludes further elucidation of the biological mechanisms underlying the protective effect of niacin status in KTR. Although many potential confounders were adjusted for in separate models, one cannot rule out the presence of additional or unmeasured confounding. Furthermore, the conclusions of this study are based on single baseline measurements, although under- and over-collection of 24-h urine is accounted for by strict protocol and sensitivity analyses, as described previously [11], and the urinary excretion of NAD^+^ catabolites is stable within individuals over time [70]. Last, the overrepresentation of Caucasian subjects from a single center may compromise the generalizability of the conclusions of this study.

## 5. Conclusions

The 24-h urinary excretion of *N*^1^-MN + 2Py is negatively associated with risk of premature all-cause mortality, and infectious mortality specifically, independent of potential confounders. As the interpretation of both *N*^1^-MN and 2Py excretion is indicated for assessment of niacin status in KTR, these findings reinforce niacin status as a potential target for nutritional strategies for improvement of long-term outcome after renal transplantation. However, future studies are warranted to address causal inferences and biological mechanisms underlying the protective effect of niacin nutritional status in KTR.

## Figures and Tables

**Table 1:** Baseline characteristics of kidney transplant recipients (KTR) across tertiles of *N*^1^-MN + 2Py excretion stratified by sex ^1,2^.

Variable	Sex-Stratified Tertiles of *N*^1^-MN + 2Py Excretion			Std. β	*p*-Value
	T1 *n* = 219	T2 *n* = 221	T3 *n* = 220		
Males, μmol/day	<181.3	181.3–261.2	>261.2	-	-
Females, μmol/day	<147.7	147.7–216.9	>216.9	-	-
Demographics					
Male, *n* (%)	126 (58)	127 (58)	126 (57)	-	-
Age, years	54.3 ± 12.6	52.3 ± 13.4	52.4 ± 12.1	−0.10	0.01
BMI, kg/m^2^	25.4 ± 4.5	26.7 ± 4.5	27.8 ± 5.1	0.19	<0.001
Body surface area, m^2^	1.9 ± 0.2	1.9 ± 0.2	2.0 ± 0.2	0.28	<0.001
Current smoker, *n* (%)	24 (12)	24 (12)	30 (15)	0.04	0.34
Alcohol consumption, g/day	1.0 (0.0–7.8)	3.2 (0.1–12.0)	5.1 (0.2–17.7)	0.18	<0.001
Nutrition					
Energy intake, kcal/day	2098 ± 619	2248 ± 718	2198 ± 576	0.06	0.17
Plasma vitamin B_6_, nmol/L	27.0 (15.0–41.0)	26.0 (17.0–42.0)	41.0 (22.0–66.0)	0.30	<0.001
Glucose homeostasis					
Glucose, mmol/L	5.2 (4.8–5.7)	5.3 (4.8–6.0)	5.3 (4.8–6.2)	0.08	0.05
HbA1c, (%)	5.8 (5.5–6.1)	5.8 (5.5–6.2)	5.8 (5.5–6.3)	−0.003	0.95
Diabetes, *n* (%)	46 (21)	51 (23)	55 (25)	0.05	0.23
Antidiabetic, *n* (%)	32 (15)	34 (15)	30 (14)	0.007	0.86
Lipid homeostasis					
Total cholesterol, mmol/L	5.2 ± 1.2	5.1 ± 1.1	5.0 ± 1.1	−0.03	0.40
LDL, mmol/L	3.0 ± 1.0	3.0 ± 0.9	3.0 ± 0.9	0.009	0.82
HDL, mmol/L	1.3 (1.1–1.7)	1.3 (1.1–1.6)	1.3 (1.1–1.6)	0.05	0.23
Triglycerides, mmol/L	1.6 (1.2–2.3)	1.7 (1.3–2.3)	1.6 (1.2–2.2)	−0.03	0.39
Statin, *n* (%)	111 (51)	122 (55)	116 (53)	−0.02	0.61
Hemodynamic					
Systolic blood pressure, mmHg	138 ± 18	135 ± 16	135 ±17	−0.08	0.05
Diastolic blood pressure, mmHg	82 ± 12	82 ± 11	83 ± 11	0.01	0.72
Mean arterial pressure, mmHg	108 ± 16	107 ± 14	107 ± 15	−0.05	0.22
Heart rate, beats per minute	68 ± 12	69 ± 13	68 ± 12	−0.006	0.87
Antihypertensive use, *n* (%)	196 (90)	193 (87)	192 (87)	−0.04	0.25
Inflammation					
Hs-CRP, mg/L	1.3 (0.5–3.5)	1.6 (0.7–4.4)	1.9 (0.9–5.6)	0.10	0.007
Renal function					
eGFR, ml/min/1.73 m^2^	44.7 ± 20.0	46.0 ± 18.5	46.6 ± 17.7	0.09	0.03
Proteinuria, *n* (%)	47 (22)	47 (21)	38 (17)	−0.06	0.14
Immunosuppressive medication					
Prednisolon dose, mg/day	7.5 (7.5–10)	7.5 (7.5–10)	7.5 (7.5–10)	0.02	0.54
Calcineurin inhibitor, *n* (%)	131 (60)	127 (58)	115 (48)	−0.05	0.23
Tacrolimus, *n* (%)	38 (17)	49 (22)	33 (15)	−0.02	0.54
Cyclosporine, *n* (%)	93 (43)	78 (35)	82 (37)	−0.03	0.46
Proliferation inhibitor, *n* (%)	172 (79)	183 (83)	193 (88)	0.10	0.01
Azathioprine, *n* (%)	35 (16)	32 (15)	45 (21)	0.04	0.36
Mycophenolic acid, *n* (%)	137 (63)	151 (68)	148 (67)	0.05	0.21
Nonimmunosuppressive medication					
Acetylsalicylic acid, *n* (%)	55 (25)	34 (15)	38 (17)	−0.08	0.03
Anticonvulsant, *n* (%)	9 (4)	4 (2)	6 (3)	−0.02	0.59
Proton pump inhibitor, *n* (%)	122 (56)	99 (45)	105 (48)	−0.09	0.03
Diuretic, *n* (%)	95 (43)	76 (34)	90 (41)	−0.05	0.21
Renal transplantation					
Time since transplantation, years	5.9 (2.6–13.4)	5.1 (1.4–10.7)	5.8 (2.4–12.2)	−0.02	0.65
Donor					
Age, years	44 (28–53)	47 (33–56)	44 (31–54)	0.002	0.97
Male, *n* (%)	108 (50)	114 (52)	104 (50)	−0.04	0.31
Post mortem status, *n* (%)	150 (69)	133 (61)	142 (66)	0.04	0.36
Primary renal disease					
Primary glomerular disease, *n* (%)	57 (26)	68 (31)	61 (28)	0.01	0.81
Glomerulonephritis, *n* (%)	15 (7)	17 (8)	18 (8)	0.06	0.14
Tubulointerstitial disease, *n* (%)	26 (12)	28 (13)	23 (11)	−0.02	0.54
Polycystic renal disease, *n* (%)	41 (19)	42 (19)	54 (25)	0.02	0.59
Dysplasia and hypoplasia, *n* (%)	10 (5)	10 (5)	8 (4)	−0.01	0.79
Renovascular disease, *n* (%)	15 (7)	8 (4)	13 (6)	−0.04	0.29
Diabetic nephropathy, *n* (%)	14 (6)	13 (6)	8 (4)	−0.03	0.46
Other or unknown cause, *n* (%)	40 (18)	35 (16)	35 (16)	−0.005	0.90

^1^ Normally distributed, skewed, and nominal data are presented as mean ± SD, median (IQR), and absolute number (percentage), respectively. ^2^ Cross-sectional associations of *N*^1^-MN + 2Py excretion with baseline variables were investigated with linear regression analyses, with adjustment for sex, of which std. β and *p*-value are presented. BMI, body mass index; eGFR, estimated glomerular filtration rate; HbA1c, hemoglobin A1c; HDL, high-density lipoprotein; hs-CRP, high-sensitivity C-reactive protein; LDL, low-density lipoprotein; *N*^1^-MN, *N*^1^-methylnicotinamide; KTR, kidney transplant recipients; std. β, standardized β; 2Py, *N*^1^-methyl-2-pyridone-5-carboxamide; 2Py/*N*^1^-MN, ratio of 2Py to *N*^1^-MN.

**Table 2:** Association of *N*^1^-MN + 2Py excretion with risk of all-cause mortality in KTR ^1^.

Model	*N*^1^-MN + 2Py Excretion (log_2_) As Continuous Variable *n* = 660		Sex-Stratified Tertiles of *N*^1^-MN + 2Py Excretion ^2^				
			T1 *n* = 219		T2 *n* = 221		T3 *n* = 220
	HR (95% CI)	*p*-Value	HR (95% CI)	*p*-Value	HR (95% CI)	*p*-Value	Reference HR
1 ^3^	0.55 (0.43–0.71)	<0.001	2.28 (1.49–3.49)	<0.001	1.52 (0.96–2.39)	0.07	1.00
2 ^4^	0.61 (0.47–0.79)	<0.001	2.03 (1.31–3.15)	0.002	1.45 (0.92–2.30)	0.11	1.00
3 ^5^	0.60 (0.46–0.78)	<0.001	2.13 (1.37–3.33)	0.001	1.51 (0.95–2.40)	0.08	1.00
4 ^6^	0.65 (0.49–0.86)	0.003	1.85 (1.17–2.94)	0.009	1.36 (0.84–2.18)	0.21	1.00
5 ^7^	0.67 (0.52–0.87)	0.003	1.93 (1.23–3.02)	0.004	1.32 (0.82–2.12)	0.25	1.00
6 ^8^	0.69 (0.53–0.90)	0.006	1.74 (1.12–2.72)	0.02	1.42 (0.90–2.25)	0.13	1.00
7 ^9^	0.70 (0.52–0.94)	0.02	1.71 (1.05–2.79)	0.03	1.39 (0.84–2.29)	0.20	1.00
Events (*n*)	143		66		46		31

^1^ The association of *N*^1^-MN + 2Py excretion with risk of all-cause mortality in KTR was investigated with Cox regression analyses, with adjustment for potential confounders. ^2^ *N*^1^-MN + 2Py excretion was <181.3, 181.3–261.2, and >261.2 μmol/day for males, and <147.7, 147.7–216.9, and >216.9 μmol/day for females in T1, T2, and T3, respectively. ^3^ Model 1: not adjusted in sex-stratified tertiles of *N*^1^-MN + 2Py excretion, adjusted for sex in continuous analyses. ^4^ Model 2: adjusted as for model 1 and for age and body surface area. ^5^ Model 3: adjusted as for model 2 and for serum hs-CRP. ^6^ Model 4: adjusted as for model 2 and for plasma vitamin B_6_. ^7^ Model 5: adjusted as for model 2 and for eGFR, proteinuria, and primary renal disease. ^8^ Model 6: adjusted as for model 2 and for use of proliferation inhibitors, acetylsalicylic acid, and proton pump inhibitors. ^9^ Model 7: adjusted as for model 2 and for intake of alcohol and energy. CI, confidence interval; eGFR, estimated glomerular filtration rate; HR, hazard ratio; hs-CRP, high-sensitivity C-reactive protein; *N*^1^-MN, *N*^1^-methylnicotinamide; KTR, kidney transplant recipients; 2Py, *N*^1^-methyl-2-pyridone-5-carboxamide.

**Table 3:** Association of *N*^1^-MN + 2Py excretion with risk of infectious mortality and noninfectious mortality in KTR ^1^.

Model	*N*^1^-MN + 2Py Excretion (log_2_) As Continuous Variable *n* = 660	
	HR (95% CI)	*p*-Value
**Infectious Mortality**		
1 ^2^	0.42 (0.27–0.66)	<0.001
2 ^3^	0.47 (0.29–0.75)	0.002
3 ^4^	0.47 (0.29–0.75)	0.002
4 ^5^	0.51 (0.31–0.86)	0.01
5 ^6^	0.54 (0.34–0.86)	0.009
6 ^7^	0.54 (0.33–0.88)	0.01
7 ^8^	0.54 (0.32–0.91)	0.02
Events (*n*)	40	
**Noninfectious Mortality**		
1 ^2^	0.62 (0.46–0.83)	0.001
2 ^3^	0.68 (0.50–0.93)	0.02
3 ^4^	0.67 (0.49–0.92)	0.01
4 ^5^	0.72 (0.51–1.00)	0.05
5 ^6^	0.74 (0.54–1.01)	0.06
6 ^7^	0.75 (0.55–1.03)	0.08
7 ^8^	0.79 (0.55–1.12)	0.18
Events (*n*)	103	

^1^ The association of *N*^1^-MN + 2Py excretion with risk of infectious mortality and noninfectious mortality in KTR was investigated with Cox regression analyses, with adjustment for potential confounders. ^2^ Model 1: adjusted for sex. ^3^ Model 2: adjusted as for model 1 and for age and body surface area. ^4^ Model 3: adjusted as for model 2 and for serum hs-CRP. ^5^ Model 4: adjusted as for model 2 and for plasma vitamin B_6_. ^6^ Model 5: adjusted as for model 2 and for eGFR, proteinuria, and primary renal disease. ^7^ Model 6: adjusted as for model 2 and for use of proliferation inhibitors, acetylsalicylic acid, and proton pump inhibitors. ^8^ Model 7: adjusted as for model 2 and for intake of alcohol and energy. CI, confidence interval; eGFR, estimated glomerular filtration rate; HR, hazard ratio; hs-CRP, high-sensitivity C-reactive protein; *N*^1^-MN, *N*^1^-methylnicotinamide; KTR, kidney transplant recipients; 2Py, *N*^1^-methyl-2-pyridone-5-carboxamide.

**Table 4:** Association of *N*^1^-MN + 2Py excretion with risk of mortality in subgroups of serum hs-CRP in KTR ^1^.

Model	Hs-CRP ≤ 3 mg/L		Hs-CRP > 3 mg/L	
	HR (95% CI)	*p*-Value	HR (95% CI)	*p*-Value
**All-Cause Mortality**				
1 ^2^	0.46 (0.34–0.64)	<0.001	0.64 (0.44–0.95)	0.03
2 ^3^	0.48 (0.34–0.68)	<0.001	0.79 (0.52–1.20)	0.27
3 ^4^	0.49 (0.35–0.68)	<0.001	0.80 (0.53–1.21)	0.29
4 ^5^	0.50 (0.35–0.72)	<0.001	0.89 (0.57–1.39)	0.61
5 ^6^	0.58 (0.42–0.82)	0.002	0.83 (0.54–1.26)	0.37
6 ^7^	0.57 (0.41–0.81)	0.002	0.83 (0.54–1.27)	0.38
7 ^8^	0.56 (0.38–0.83)	0.003	0.90 (0.57–1.42)	0.64
Events (*n*)	81		62	
**Infectious Mortality**				
1 ^2^	0.35 (0.20–0.60)	<0.001	0.60 (0.28–1.32)	0.21
2 ^3^	0.38 (0.21–0.67)	0.001	0.71 (0.30–1.65)	0.43
3 ^4^	0.39 (0.22–0.67)	0.001	0.70 (0.30–1.64)	0.41
4 ^5^	0.38 (0.21–0.70)	0.002	0.97 (0.39–2.43)	0.95
5 ^6^	0.47 (0.27–0.83)	0.009	0.79 (0.34–1.84)	0.58
6 ^7^	0.45 (0.25–0.81)	0.008	0.77 (0.31–1.89)	0.56
7 ^8^	0.40 (0.20–0.78)	0.008	0.79 (0.34–1.81)	0.58
Events (*n*)	25		15	
**Noninfectious Mortality**				
1 ^2^	0.53 (0.36–0.78)	0.001	0.66 (0.42–1.02)	0.06
2 ^3^	0.54 (0.36–0.83)	0.005	0.82 (0.51–1.31)	0.41
3 ^4^	0.54 (0.36–0.83)	0.005	0.84 (0.52–1.34)	0.46
4 ^5^	0.57 (0.37–0.90)	0.02	0.87 (0.52–1.44)	0.58
5 ^6^	0.65 (0.42–0.98)	0.04	0.84 (0.51–1.37)	0.48
6 ^7^	0.64 (0.42–0.97)	0.04	0.82 (0.51–1.31)	0.41
7 ^8^	0.66 (0.42–1.05)	0.08	0.95 (0.55–1.65)	0.87
Events (*n*)	56		47	

^1^ The association of *N*^1^-MN + 2Py excretion with risk of all-cause mortality, infectious mortality, and noninfectious mortality in KTR was investigated with Cox regression analyses, with adjustment for potential confounders. ^2^ Model 1: adjusted for sex. ^3^ Model 2: adjusted as for model 1 and for age and body surface area. ^4^ Model 3: adjusted as for model 2 and for serum hs-CRP. ^5^ Model 4: adjusted as for model 2 and for plasma vitamin B_6_. ^6^ Model 5: adjusted as for model 2 and for eGFR, proteinuria, and primary renal disease. ^7^ Model 6: adjusted as for model 2 and for use of proliferation inhibitors, acetylsalicylic acid, and proton pump inhibitors. ^8^ Model Model 7: adjusted as for model 2 and for intake of alcohol and energy. CI, confidence interval; eGFR, estimated glomerular filtration rate; HR, hazard ratio; hs-CRP, high-sensitivity C-reactive protein; *N*^1^-MN, *N*^1^-methylnicotinamide; KTR, kidney transplant recipients; 2Py, *N*^1^-methyl-2-pyridone-5-carboxamide.

**Table 5:** Urinary excretion of *N*^1^-MN + 2Py*, N*^1^-MN, 2Py and 2Py/*N*^1^-MN, and dietary intake of niacin equivalents across tertiles of *N*^1^-MN + 2Py excretion stratified by sex in KTR ^1,2^.

Variable	Tertiles of Sex-Stratified *N*^1^-MN + 2Py Excretion			Std. β	*p*-Value
	T1 *n* = 219	T2 *n* = 221	T3 *n* = 220		
Urinary excretion					
*N*^1^-MN + 2Py, μmol/day	131.5 (110.5–150.9)	203.6 (181.5–225.6)	313.8 (274.2–382.8)	-	-
*N*^1^-MN, μmol/day	14.7 (10.9–19.4)	21.5 (17.6–27.7)	34.7 (26.1–45.3)	0.74	<0.001
2Py, μmol/day	114.5 (94.0–131.6)	178.2 (155.6–198.3)	280.0 (242.1–340.4)	0.99	<0.001
2Py/*N*^1^-MN	7.8 (6.0–9.7)	8.3 (6.5–10.4)	8.8 (6.4 –11.5)	−0.16	<0.001
Dietary intake					
Niacin equivalents intake, mg/day	33.1 ± 8.5	36.6 ± 9.7	36.9 ± 9.1	0.18	<0.001

^1^ Normally distributed, skewed, and nominal data are presented as mean ± SD, median (IQR) and absolute number (percentage), respectively. ^2^ Cross-sectional associations of urinary excretion of *N*^1^-MN, 2Py and 2Py/*N*^1^-MN, and dietary intake of niacin equivalents with *N*^1^-MN + 2Py excretion were investigated with linear regression analyses, with adjustment for sex, of which std. β and *p*-value are presented. *N*^1^-MN, *N*^1^-methylnicotinamide; KTR, kidney transplant recipients; std. β, standardized β; 2Py, *N*^1^-methyl-2-pyridone-5-carboxamide; 2Py/*N*^1^-MN, ratio of 2Py to *N*^1^-MN.

**Table 6:** Association of urinary excretion of *N*^1^-MN, 2Py and 2Py/*N*^1^-MN, and dietary intake of niacin equivalents with risk of all-cause mortality in KTR ^1^.

Model	Urinary Excretion						Dietary Intake Niacin Equivalents, mg/day ^2^	
	*N*^1^-MN, µmol/day		2Py, µmol/day		2Py/*N*^1^-MN			
	HR (95% CI)	*p*-Value	HR (95% CI)	*p*-Value	HR (95% CI)	*p*-Value	HR (95% CI)	*p*-Value
1 ^3^	0.53 (0.43–0.65)	<0.001	0.59 (0.47–0.75)	<0.001	1.06 (1.02–1.10)	0.003	0.58 (0.42–0.81)	0.001
2 ^4^	0.57 (0.46–0.72)	<0.001	0.65 (0.51–0.84)	0.001	1.06 (1.02–1.10)	0.005	0.61 (0.43–0.87)	0.006
3 ^5^	0.58 (0.47–0.73)	<0.001	0.64 (0.49–0.82)	0.001	1.05 (1.01–1.10)	0.02	0.65 (0.46–0.93)	0.02
4 ^6^	0.61 (0.48–0.77)	<0.001	0.69 (0.53–0.91)	0.009	1.06 (1.01–1.10)	0.01	0.65 (0.45–0.93)	0.02
5 ^7^	0.73 (0.57–0.92)	0.009	0.69 (0.53–0.89)	0.004	1.00 (0.95–1.04)	0.85	0.69 (0.48–0.98)	0.04
6 ^8^	0.63 (0.50–0.78)	<0.001	0.73 (0.57–0.94)	0.02	1.06 (1.02–1.10)	0.007	0.64 (0.45–0.91)	0.02
7 ^9^	0.64 (0.50–0.82)	<0.001	0.74 (0.56–0.98)	0.04	1.07 (1.02–1.12)	0.006	0.77 (0.43–1.38)	0.38

^1^ The association of urinary excretion of *N*^1^-MN, 2Py and 2Py/*N*^1^-MN, and dietary intake of niacin equivalents with risk of all-cause mortality in KTR was investigated with Cox regression analyses, with adjustment for potential confounders. ^2^ HRs per 15 mg increase in dietary intake of niacin equivalents are presented. ^3^ Model 1: adjusted for sex. ^4^ Model 2: adjusted as for model 1 and for age and body surface area. ^5^ Model 3: adjusted as for model 2 and for serum hs-CRP. ^6^ Model 4: adjusted as for model 2 and for plasma vitamin B_6_. ^7^ Model 5: adjusted as for model 2 and for eGFR, proteinuria, and primary renal disease. ^8^ Model 6: adjusted as for model 2 and for use of proliferation inhibitors, acetylsalicylic acid, and proton pump inhibitors. ^9^ Model 7: adjusted as for model 2 and for intake of alcohol and energy. CI, confidence interval; eGFR, estimated glomerular filtration rate; HR, hazard ratio; hs-CRP, high-sensitivity C-reactive protein; *N*^1^-MN, *N*^1^-methylnicotinamide; KTR, kidney transplant recipients; 2Py, *N*^1^-methyl-2-pyridone-5-carboxamide; 2Py/*N*^1^-MN, ratio of 2Py to *N*^1^-MN.

## Supplementary Materials

The following is available online at https://www.mdpi.com/2072-6643/12/7/2059/s1, Table S1: Association of *N*^1^-MN + 2Py excretion with risk of cardiovascular mortality, malignant mortality, and other (miscellaneous) mortality in KTR, Table S2: Nonlinearity of associations of *N*^1^-MN + 2Py excretion with risk of all-cause mortality and infectious mortality in KTR, Table S3: Association of *N*^1^-MN + 2Py excretion with risk of all-cause mortality and infectious mortality in a subgroup of KTR that died during >3.17 years of follow-up.
