# Supplementary material for: Urinary Excretion of N1-Methylnicotinamide and N1-Methyl-2-Pyridone-5-Carboxamide and Mortality in Kidney Transplant Recipients
Source: Nutrients. 2020 Jul 10;12(7):2059. doi: 10.3390/nu12072059 (PMC7400946; doi:10.3390/nu12072059)
Supplement: Supplementary file 1 [file nutrients-12-02059-s001.pdf]

Supplementary Table S1. Association of *N*[1]-MN + 2Py excretion with risk of cardiovascular mortality, malignant mortality, and other (miscellaneous) mortality in KTR [1].

Model	*N*[1]-MN + 2Py Excretion (log_2) As Continuous Variable *n* = 660	
	HR (95% CI)	*p*-Value
**Cardiovascular Mortality**		
1 [2]	0.69 (0.46–1.03)	0.07
2 [3]	0.73 (0.48–1.12)	0.15
3 [4]	0.73 (0.48–1.12)	0.15
4 [5]	0.77 (0.49–1.23)	0.77
5 [6]	0.79 (0.51–1.22)	0.29
6 [7]	0.81 (0.53–1.24)	0.34
7 [8]	0.92 (0.58–1.46)	0.73
Events (*n*)	56	
**Malignant Mortality**		
1 [2]	0.50 (0.29–0.87)	0.01
2 [3]	0.54 (0.29–0.98)	0.04
3 [4]	0.49 (0.27–0.91)	0.02
4 [5]	0.45 (0.24–0.86)	0.02
5 [6]	0.57 (0.30–1.06)	0.08
6 [7]	0.57 (0.31–1.05)	0.07
7 [8]	0.56 (0.27–1.14)	0.11
Events (*n*)	26	
**Other (Miscellaneous) Mortality**		
1 [2]	0.60 (0.31–1.16)	0.13
2 [3]	0.79 (0.39–1.58)	0.50
3 [4]	0.80 (0.39–1.61)	0.52
4 [5]	1.11 (0.51–2.43)	0.79
5 [6]	0.87 (0.43–1.74)	0.68
6 [7]	0.92 (0.46–1.85)	0.81
7 [8]	0.73 (0.32–1.65)	0.45
Events (*n*)	21	

[1] The association of *N*[1]-MN + 2Py excretion with risk of cardiovascular mortality, malignant mortality, and other (miscellaneous) mortality in KTR was investigated with Cox regression analyses, with adjustment for potential confounders. [2] Model 1: adjusted for sex. [3] Model 2: adjusted as for model 1 and for age and body surface area. [4] Model 3: adjusted as for model 2 and for serum hs-CRP. [5] Model 4: adjusted as for model 2 and for plasma vitamin B_6. [6] Model 5: adjusted as for model 2 and for eGFR, proteinuria, and primary renal disease. [7] Model 6: adjusted as for model 2 and for use of proliferation inhibitors, acetylsalicylic acid, and proton pump inhibitors. [8] Model 7: adjusted as for model 2 and for intake of alcohol and energy. CI, confidence interval; eGFR, estimated glomerular filtration rate; HR, hazard ratio; hs-CRP, high-sensitivity C-reactive protein; *N*[1]-MN, *N*[1]-methylnicotinamide; KTR, kidney transplant recipients; 2Py, *N*[1]-methyl-2-pyridone-5-carboxamide.

Supplementary Table S2. Nonlinearity of associations of N^1-MN + 2Py excretion with risk of all-cause mortality and infectious mortality in KTR [1].

	Urinary Excretion of N^1-MN + 2Py	
**Model**	**Quadratic term**	**Cubic term**
	***p*-Value**	***p*-Value**
	**All-Cause Mortality**	
1 [3]	0.39	0.39
2 [4]	0.22	0.24
3 [5]	0.21	0.22
4 [6]	0.20	0.22
5 [7]	0.31	0.47
6 [8]	0.31	0.33
7 [9]	0.33	0.38
Events (*n*)	143	
	**Infectious Mortality**	
1 [2]	0.23	0.48
2 [3]	0.33	0.37
3 [4]	0.34	0.38
4 [5]	0.20	0.22
5 [6]	0.54	0.58
6 [7]	0.31	0.36
7 [8]	0.18	0.21
Events (*n*)	40	

[1] Nonlinearity of associations of N^1-MN + 2Py excretion with risk of all-cause mortality and infectious mortality in KTR was investigated with Cox regression analyses by including the quadratic and cubic terms of N^1-MN + 2Py excretion, with adjustment for potential confounders. [2] Model 1: adjusted for sex. [3] Model 2: adjusted as for model 1 and for age and body surface area. [4] Model 3: adjusted as for model 2 and for serum hs-CRP. [5] Model 4: adjusted as for model 2 and for plasma vitamin B_6. [6] Model 5: adjusted as for model 2 and for eGFR, proteinuria, and primary renal disease. [7] Model 6: adjusted as for model 2 and for use of proliferation inhibitors, acetylsalicylic acid, and proton pump inhibitors. [8] Model 7: adjusted as for model 2 and for intake of alcohol and energy. CI, confidence interval; eGFR, estimated glomerular filtration rate; hs-CRP, high-sensitivity C-reactive protein; N^1-MN, N^1-methylnicotinamide; KTR, kidney transplant recipients; 2Py, N^1-methyl-2-pyridone-5-carboxamide.

Supplementary Table S3 Association of *N*[1]-MN + 2Py excretion with risk of all-cause mortality and infectious mortality in a subgroup of KTR that died during >3.17 years of follow-up [1].

**Model**	**N^1-MN + 2Py Excretion ($\log_2$) As Continuous Variable n = 660**	
	**HR (95% CI)**	***p*-Value**
	**All-Cause Mortality**	
1 [2]	0.49 (0.34–0.70)	<0.001
2 [3]	0.56 (0.39–0.82)	0.002
3 [4]	0.56 (0.39–0.82)	0.002
4 [5]	0.60 (0.40–0.90)	0.01
5 [6]	0.60 (0.41–0.88)	0.009
6 [7]	0.62 (0.42–0.91)	0.01
7 [8]	0.62 (0.42–0.91)	0.03
Events (*n*)	71	
	**Infectious Mortality**	
1 [2]	0.26 (0.14–0.48)	<0.001
2 [3]	0.28 (0.14–0.55)	<0.001
3 [4]	0.28 (0.14–0.56)	<0.001
4 [5]	0.27 (0.13–0.55)	<0.001
5 [6]	0.28 (0.14–0.57)	<0.001
6 [7]	0.33 (0.16–0.67)	0.002
7 [8]	0.36 (0.18–0.74)	0.006
Events (*n*)	23	

[1] The association of *N*[1]-MN + 2Py excretion with risk of all-cause mortality and infectious mortality was investigated in with Cox regression analyses, with adjustment for potential confounders. [2] Model 1: adjusted for sex. [3] Model 2: adjusted as for model 1 and for age and body surface area. [4] Model 3: adjusted as for model 2 and for serum hs-CRP. [5] Model 4: adjusted as for model 2 and for plasma vitamin B_6. [6] Model 5: adjusted as for model 2 and for eGFR, proteinuria, and primary renal disease. [7] Model 6: adjusted as for model 2 and for use of proliferation inhibitors, acetylsalicylic acid, and proton pump inhibitors. [8] Model 7: adjusted as for model 2 and for intake of alcohol and energy. CI, confidence interval; eGFR, estimated glomerular filtration rate; HR, hazard ratio; hs-CRP, high-sensitivity C-reactive protein; *N*[1]-MN, *N*[1]-methylnicotinamide; KTR, kidney transplant recipients; 2Py, *N*[1]-methyl-2-pyridone-5-carboxamide.
